# Voluntary activation and twitch potentiation of the elbow flexors across supinated, neutral, and pronated forearm orientations

**DOI:** 10.14814/phy2.13560

**Published:** 2018-01-15

**Authors:** Sienna Kohn, Rowan R. Smart, Jennifer M. Jakobi

**Affiliations:** ^1^ School of Health and Exercise Sciences Healthy Exercise and Aging Lab Group University of British Columbia Okanagan Kelowna British Columbia Canada

**Keywords:** Biceps brachii, contractile properties, force, strength

## Abstract

Elbow flexion force depends on forearm orientation with supinated and neutral being stronger than pronated. The purpose of this study was to assess the influence of forearm orientation on voluntary activation (VA), postactivation potentiation (PAP), and twitch properties. Eleven males (23 ± 3 years) performed isometric elbow flexion maximal voluntary contractions (MVC) in supinated, neutral, and pronated forearm orientations with supramaximal stimulation to the biceps brachii muscle belly before, during, and after the MVC. MVC and VA were higher in supinated (213.6 ± 49.6 N; 93.0 ± 5.2%) and neutral (243.6 ± 48.0 N; 96.1 ± 3.2%) compared with pronated (113.6 ± 21.3 N; 70.9 ± 20.4%) (*P* < 0.05), while PAP did not differ across the three orientations (71.6 ± 42.2%) (*P* > 0.05). In the rested state, pronated peak tension (PT) was less compared with supinated (42%). In the potentiated state, pronated PT was less than supinated (50%) and neutral (53%) (*P* < 0.05). Reduced strength in the pronated orientation is partially attributed to reduced drive; however, reductions in peak tension indicate that there also is a mechanical disadvantage when the forearm is placed into a pronated orientation, and this does not alter PAP.

## Introduction

A maximal voluntary contraction (MVC) can act as a conditioning stimulus to produce an acute enhancement of force, known as postactivation potentiation (PAP) (Vandervoort et al. 1983). The extent of PAP is dependent on the amount of muscle activation, where higher levels of activation increase force output and culminate in greater PAP following the MVC through Ca^2+^‐induced phosphorylation of the myosin regulatory light chains (Sweeny et al. 1993; Rassier and MacIntosh 2000; Sale 2002; de Tombe et al. 2006; Miyamoto et al. 2010). Increased force capacity in the potentiated state also contributes to faster twitch contractile times and rates, albeit somewhat controversial faster relaxation times in whole human muscle (Hamada et al. 2000; Baudry and Duchateau 2007; Baudry et al. 2008).

To ensure that the conditioning MVC is maximal in order to achieve high levels of PAP, the twitch interpolation technique can be applied to quantify the degree of voluntary activation (VA) (Allen et al. 1995; Jakobi and Rice 2002). Greater MVC force output results in a smaller interpolated twitch and higher VA (Arampatzis et al. 2007; Kooistra et al. 2007; Taylor 2009; Huang et al. 2010). What remains unknown is whether position‐dependent force production of the elbow flexors, whereby the pronated orientation is ~40% weaker than supinated and neutral (Brown et al. 2010), is due to reductions in VA of the biceps brachii in pronated, and whether PAP occurs in the pronated orientation when MVC is considerably reduced. Therefore, the purpose of this study was to quantify VA, PAP, and twitch contractile properties across supinated, neutral, and pronated forearm orientations. It was hypothesized that the pronated orientation would have reduced VA, PAP, and twitch properties compared to supinated and neutral.

## Materials and Methods

### Participants

Eleven right‐hand dominant males (23 ± 3 years, 175.6 ± 8.2 cm, 72.9 ± 7.5 kg) volunteered to participate in this study. Ethics approval was gained from the University of British Columbia Okanagan Behavioural Research Ethics Board, and informed written consent was obtained from each participant prior to commencing the study. Exclusion criteria included: (1) active tendinopathy, (2) injury/orthopedic surgery to right arm or shoulder in the prior 6 months, (3) systemic diseases affecting collagenous tissue, (4) participation in high levels of upper body strength training, (5) history of training in fine motor tasks (e.g., musician), and/or (6) nerve damage to the right arm.

### Experimental setup

Participants were seated upright in a custom dynamometer chair with knees and hips positioned and maintained at 90°, the dominant (right) elbow was flexed to 110° (full extension being 180°) and placed into a supportive mold that cupped the elbow into a constant position. The shoulder was abducted 10° and flexed forward at 15° for each orientation. The forearm apparatus through consistent positioning of the elbow with the hand gripping the manipulandum was maintained constant across supinated, neutral, and pronated orientations. The force transducer was located immediately below the hand (MLP‐150 linear force transducer, 68 kg, 266 V sensitivity, Transducer Techniques, Temecula, CA). As previously described (Harwood et al. 2010), the forearm was placed into a supinated, neutral, or pronated orientation by rotating the manipulandum in the direction of the desired orientation. Force was displayed in real time on a 52‐cm monitor located 1 m in front of the participant for visual feedback. Force signals were amplified (100×) (Coulbourn Electronics, Allentown, PA), sampled at 2381 Hz and converted from analog to digital using a Power 1401 (Cambridge Electronic Design [CED], Cambridge, England), and stored for offline analysis using Spike 2 version 7 (CED, Cambridge, England). Two 4 cm × 4 cm carbon–carbon stimulation electrodes coated in electrode gel were placed proximally and distally on the biceps brachii (BB) muscle belly to evoke supramaximal twitches.

### Protocol

Supramaximal stimulation intensity was established for the supinated, neutral, and pronated orientations in a randomized order. Stimulation (100 *μ*sec pulse width, DS7AH, Digitimer Ltd., Welwyn Garden City, UK) to the BB was progressively increased until a plateau in twitch force amplitude occurred and then increased a further 10% to achieve supramaximal intensity. In randomized order, participants performed two to three 5‐sec MVCs in each orientation with twitches applied at rest, during the MVC, and post‐MVC at 1‐sec intervals to measure VA and induce PAP. A rest period of 2–3 min between each contraction was given and 3–5 min rest between orientations. The highest MVC value of the 2–3 trials was used for subsequent force and twitch analysis.

### Data analysis

#### Force

VA during the MVCs was calculated using the twitch interpolation technique with the following formula: VA (%) = (1 − [interpolated twitch/resting twitch]) × 100 (Allen et al. 1995; Jakobi and Rice 2002; Simpson et al. 2016). Postactivation potentiation was calculated as the percentage increase in peak twitch torque from the resting (using the twitch directly prior to MVC) to potentiated twitch.

#### Twitch properties

Twitch properties of peak tension, time to peak tension (TPT), half relaxation time (HRT), peak rise, and peak fall were measured. Peak tension was calculated as the highest tension (peak) of the twitch from the baseline. TPT was calculated as time (msec) from the onset of the evoked force to the peak tension of the twitch and HRT as the time for the twitch to relax half of maximal amplitude (msec). Peak rise (N/msec) and peak fall (N/msec) were measured as the first derivative of the force signal and reported for the rate of rise and fall of the twitch.

### Statistical analysis

Using Statistical Package for Social Sciences (SPSS) version 24 (IBM, Armonk, NY), a one‐way ANOVA was used to compare VA, PAP, and MVC across supinated, neutral, and pronated forearm orientations. To assess contractile properties, a 2 (time: resting twitch, potentiated twitch) × 3 (orientation: supinated, neutral, and pronated) repeated measures ANOVA was applied. Significant interactions were assessed with a Tukey's post hoc test, and the alpha level was set at *P* < 0.05. Values are reported as mean ± SD.

## Results

In the pronated orientation, MVC (113.6 ± 21.3 N) and VA (70.9 ± 20.4%) were less compared with supinated (213.6 ± 49.6 N; 93.0 ± 5.2%) and neutral (243.6 ± 48.0 N; 96.1 ± 3.2%) (*P* < 0.05), which did not differ from each other (*P* > 0.05). Twitch potentiation occurred in all orientations, but did not differ across supinated (70.7 ± 33.8%), neutral (86.1 ± 40.4%), and pronated (58.1 ± 52.3%) (*P* > 0.05) orientations. To ensure that differences did not arise from the contractions inducing potentiation in the resting twitches, they were compared within an orientation across the three contractions. The amplitude of the resting twitch peak tensions were highly correlated within supinated (*R* ^2^ = 0.90), neutral (*R* ^2^ = 0.92), and pronated (*R* ^2^ = 0.90) orientations. To further explore this, the percent difference was calculated as [% diff = ([first resting twitch − second resting twitch]/second resting twitch) × 100] between resting twitches prior to the first MVC, second MVC, and third MVC. The resting twitches were similar between the first and second (1%, −0.05%, 0.4%), first to third (−3.4%, 1.8%, 3.0%), and second to third (−4.1%, 1.9%, 2.7%) twitches in the supinated, neutral, and pronated orientations, respectively. These data indicate that there was sufficient time to allow dissipation of potentiation between contractions.

There was a time by orientation interaction observed for twitch contractile properties of peak tension (*F* = 6.98, *P* < 0.01), TPT (*F* = 8.078, *P* < 0.01), peak rate of rise (*F* = 3.54, *P* < 0.05), and peak rate of fall (*F* = 3.48, *P* < 0.05) (Table 1). HRT had no time by orientation interaction (*F* = 0.78, *P* > 0.05) or main effects of time (*F* = 0.89, *P* > 0.05) or orientation (*F* = 2.74, *P* > 0.05). Resting twitch peak tension was less in pronated compared with supinated (*P* < 0.05), and potentiated twitch peak tension was less in pronated compared with supinated and neutral (*P* < 0.05). Time to peak tension decreased from the resting to potentiated twitch in pronated (*P* < 0.05). TPT of the resting twitch was less in supinated compared to pronated (*P* < 0.05). Peak rise and fall of the potentiated twitch were slower in pronated compared to neutral (*P* < 0.05). Peak fall was faster in the potentiated twitch compared to the resting twitch for supinated and neutral (*P* < 0.05), and peak fall was slower in pronated compared with supinated and neutral for the potentiated twitch (*P* < 0.05) (Table 1).

**Table 1:** Twitch contractile properties of resting and potentiated twitches across the three forearm orientations

Twitch properties	Supinated		Neutral		Pronated	
	Resting	Potentiated	Resting	Potentiated	Resting	Potentiated
Peak tension (N)	22.1 ± 7.7	34.9 ± 8.1^a^	18.2 ± 5.7	32.6 ± 8.1^a^	14.9 ± 6.4^c^	20.9 ± 6.2^a^, ^c^, ^b^
TPT (msec)	41.3 ± 6.6	42.8 ± 7.7	46.2 ± 13.6	42.1 ± 11.8	59.4 ± 12.8^c^	40.5 ± 11.7^a^
HRT (msec)	42.2 ± 23.0	37.3 ± 21.6	50.9 ± 13.4	43.1 ± 17.6	55.4 ± 12.0	58.3 ± 14.6
Peak rise (N/msec)	1.3 ± 0.6	2.0 ± 0.9^a^	1.7 ± 1.1	2.7 ± 1.2^a^	1.0 ± 0.5	1.5 ± 0.7^a^, ^b^
Peak fall (N/msec)	−0.8 ± 0.3	−1.4 ± 0.4^a^	−0.9 ± 0.6	−1.5 ± 0.9^a^	−0.5 ± 0.2	−0.6 ± 0.3^c^, ^b^

TPT, time to peak tension; HRT, half relaxation time; N, Newton; significant at alpha 0.05.

a Differs from resting twitch.

b Differs from neutral orientation in same condition.

c Differs from supinated orientation in same condition.

## Discussion

In this study, MVC, VA, PAP, and twitch contractile properties of the BB were measured in the supinated, neutral, and pronated forearm orientations for isometric elbow flexion. In support of the hypothesis, MVC and VA were less in the pronated orientation compared with supinated and neutral, which did not differ. However, the nonsignificant difference in PAP across the three orientations was not expected. Peak tension was less in pronated compared with supinated at rest and less than both supinated and neutral in the potentiated state. Overall, potentiated twitches were higher and had a faster peak rise and peak fall compared with the resting twitch. Force production in the pronated orientation is reduced and this likely arises from reductions in descending drive as suggested by the decline in VA. As well, reduced force in pronated likely occurs from a mechanical disadvantage that is induced through rotation of the forearm; evident in the decrease in peak tension, in the pronated position.

We have previously reported that MVC is lower in pronated compared with supinated and neutral orientations (Brown et al. 2010), and this study suggests that this reduction in maximal strength occurs through a decrease in descending drive to the BB in the pronated orientation. This is marked by the reduced VA in pronated orientation and supported by previous observations of lower motor evoked potentials in pronated compared with supinated and neutral orientations (Mogk et al. 2014; Forman et al. 2016; Nuzzo et al. 2016). Voluntary activation calculated using the twitch interpolation technique does not offer insight into where descending drive to the motoneurons fails or the input–output relationship of the motoneuron pool (Herbert and Gandevia 1999; Kooistra et al. 2007; Taylor 2009); however, it does offer evidence that drive to the BB is reduced in the pronated orientation. This decrease in drive might arise from central sources (Mogk et al. 2014; Forman et al. 2016; Nuzzo et al. 2016), but peripheral sources, such as inhibition from the brachioradialis (Barry et al. 2008) leading to increased motor unit recruitment thresholds in the short head of the BB for the pronated forearm orientation (Harwood et al. 2010), should not be ignored. The potential contribution of peripheral sources to reduced force of the pronated orientation is supported by lower peak tension of the resting and potentiated twitches in the electrically evoked contractions.

The similar level of PAP in pronated relative to neutral and supinated orientations was not expected, as we hypothesized that the reduced MVC would result in lower levels of PAP in pronated. Despite the lower peak tension of the twitches in the pronated orientation, the relative increase in peak tension from resting to potentiated was similar to that of supinated and neutral. The lack of significant differences in PAP across orientations might arise from the high level of variability within each orientation as seen by the SD of supinated (33.8%), neutral (40.4%), and pronated (52.3%) orientations. Although the variability was high, it aligns with the large range of PAP reports in the literature (Baudry and Duchateau 2007; Hamada et al. 2000; : Vandervoort et al. 1983). Despite the lower VA and MVC in the pronated orientation, this did not affect the ability of the twitches to potentiate. This suggests that the mechanisms that induce PAP through phosphorylation of the regulatory light chains are unaffected by forearm orientation and that MVC is sufficient to induce PAP, albeit VA is substantially lower. As force was less in the involuntary contraction, as highlighted in the ~70% reduction in peak tension in pronated relative to neutral and supinated orientations, there are factors beyond descending drive inhibiting force production in the pronated orientation.

Muscle length has been shown to influence VA, with VA being greater at short compared with long muscle lengths (Suter and Herzog 1997; Kluka et al. 2015). As elbow flexion angle increases, the length of the BB increases (Ismail and Ranatunga 1978; Doheny et al. 2008). Due to the insertion of the distal BB tendon, as the forearm is rotated into the pronated orientation and the radius is rotated medially (Koch and Tillman 1995), there is likely a lengthening of the muscle as the tendon articulates over the radial head. Thus, rotation of the forearm into pronation might impart lengthening that may coincide with moving along the force–length curve and culminate in reduced force production in the pronated orientation. The length of the BB was not recorded during contraction due to the limitations of gaining ultrasound images with stimulation pads placed over the BB, thus we can only speculate on differences in muscle length between orientations. Torque production is dependent on the muscle's moment arm and prior studies have shown increases in BB moment arm up to elbow flexion angles of 100° (Murray et al. 1995), and with increasing force (Akagi et al. 2012). However, the lever arm, due to the fixed position of the forearm and elbow joint, remained identical across orientations. Previously reported moment arms of the BB were also found to be similar across supinated, neutral, and pronated forearm orientations at 110° of flexion (Murray et al. 1995). Although it is unlikely that moment arm is a key contributor to reduced force in the pronation position, future studies should quantify the combined influence of BB muscle length and tendon moment arm across forearm orientations.

In conclusion, greater elbow flexor strength in supinated and neutral orientations compared with pronated likely arises from both reductions in descending drive and mechanics of force production. This might be consequential to position‐dependent alterations in muscle and tendon. To tease out these mechanical and neural effects, and ascertain the location of altered drive, transcranial magnetic stimulation and transmastoid stimulation should be used for identification of cortical and spinal excitability in combination with ultrasonography to measure muscle and tendon mechanics.

## Conflict of Interest

The authors declare no conflict of interest.
